# Interpretation and Prognostic Value of Positron Emission Tomography-Computed Tomography After Induction Chemotherapy With or Without Radiation in IIIA-N2 Non-small Cell Lung Cancer Patients Who Receive Curative Surgery

**DOI:** 10.1097/MD.0000000000000955

**Published:** 2015-06-19

**Authors:** Sung Hwan Kim, Jong Hoon Lee, Guk Jin Lee, Songmi Jeong, Yoo-Kang Kwak, Hoon-Kyo Kim, Deog Gon Cho, Young Ha Park, Mina Yu, Sei Chul Yoon

**Affiliations:** From the Department of Radiation Oncology (SHK, JHL, SMJ, YKK); Internal Medicine (HKK); Thoracic Surgery (DGC); Nuclear Medicine (YHP), St. Vincent's Hospital, College of Medicine, The Catholic University of Korea, Republic of Korea and Radiation Oncology (MNY, SCY); Internal Medicine (GJL), Bucheon St. Mary's Hospital, College of Medicine, The Catholic University of Korea, Republic of Korea.

## Abstract

We evaluate the correlation of clinical staging on positron emission tomography-computed tomography (PET-CT) and pathologic staging and the prognostic value of PET-CT after induction chemotherapy in patients with locally advanced nonsmall cell lung cancer (NSCLC).

We analyzed 42 cases of clinical stage IIIA-N2 NSCLC who receive 2 to 4 cycles of preoperative chemotherapy with or without radiation followed by curative resection. The maximum standard uptake value (SUVmax) of the suspected lesion on PET-CT was recorded. PET-CT findings after induction chemotherapy were compared with those of initial PET-CT and pathology after surgery.

The accuracy of PET-CT in restaging of the primary tumor after induction chemotherapy was 50.0%. Eighteen (42.8%) of 42 patients were underestimated ycT stage, and 3 (7.1%) of 42 patients was overestimated ycT stage by PET-CT scan. The accuracy of PET-CT in restaging of the nodal disease was 71.4%. Six (14.3%) of 42 patients were underestimated ycN stage, and 6 (14.3%) of 42 patients were overestimated ycN stage as compared with pathologic staging. The 2-year overall survival (OS) and relapse-free survival (RFS) rate were 68.5% and 40.9%, respectively. Complete responders (ycT0N0M0) on PET-CT after induction chemotherapy had a significantly longer RFS time than did incomplete responders (28.3 vs 9.1 months, *P* = 0.021).

Complete response on PET-CT after induction chemotherapy with or without radiation was a good prognosticator for RFS in stage IIIA-N2 NSCLC patients who received surgery. However, response evaluation on PET-CT after induction chemotherapy should be interpreted with caution due to its unacceptable accuracy.

## INTRODUCTION

Lung cancer is divided into 2 distinct subtypes small-cell lung cancer and nonsmall cell lung cancer (NSCLC). NSCLC accounts for approximately 80% of all bronchogenic malignancies, and about one third of patients with NSCLC are diagnosed as locally advanced disease at their initial findings.^1,2^ The optimal management for patients with clinical stage IIIA-N2 NSCLC has not been clearly defined. For these patients, National Comprehensive Cancer Network guideline recommends definitive concurrent chemoradiotherapy or induction chemotherapy with or without radiation followed by surgery.^3^ Induction preoperative chemotherapy with or without radiotherapy followed by surgery resulted in cure rates of 25% to 35% at 3 years for stage IIIA-N2 NSCLC.^4,5^

Accurate clinical staging of NSCLC is essential for guiding clinicians to a tailored therapeutic decision. Clinicians usually use conventional chest computed-tomography (CT) scan for clinical staging of NSCLC. However, the accuracy and sensitivity of mediastinal nodal staging of CT scan have been disappointing. Positron emission tomography (PET) in conjunction with CT has shown to be a complimentary tool for the exact diagnosis and staging of NSCLC.^6^ In De Leyn et al,^7^ the sensitivity, specificity, and accuracy of PET-CT were 77%, 92%, and 83%, respectively, and recent trials have proposed the possibility that measuring standard uptake value of PET-CT would be a prognostic marker for survival.^8^ However, the accuracy and prognostic value of PET-CT after induction treatment in NSCLC has been poorly understood. Thus, we have retrospectively analyzed the correlation of PET-CT and surgical staging and the prediction of prognosis after induction chemotherapy and surgery in patients with locally advanced NSCLC.

## MATERIALS AND METHODS

### Patients

We enrolled 42 consecutive patients with histologically confirmed IIIA-N2 NSCLC who had received preoperative induction chemotherapy between March 2006 and February 2014. The inclusion criteria for the study were as follows: histologically confirmed stage IIIA-N2 NSCLC; resectable disease after induction chemotherapy; no evidence of distant metastasis; available PET-CT scans of pre- and postinduction chemotherapy; and (5) no history of malignancy other than nonmelanoma skin cancer. Of the 42 patients, 14 patients received concurrent radiotherapy of 45 to 54 Gy with induction chemotherapy.

Clinical staging work-up included chest CT, brain MRI, and PET-CT scans from the base of the skull to the midfemoral region were performed before and 1 month after induction chemotherapy, respectively. Histological diagnosis that was obtained by mediastinoscopy or bronchoscopy was mandatory. All patients were followed every 3 months for the first 2 years and every 6 months thereafter. Evaluation consisted of clinical examination, complete blood counts, and chest X-ray at each visit. Chest CT was scanned every 6 months after surgery. Institutional review board approval was obtained before the study.

### Acquisition and Interpretation of PET-CT Image

Patients were instructed to fast for 6 hours before the PET-CT scan. Three hundred seventy to 555 MBq of fluorodeoxyglucose (FDG) was injected intravenously and scanning began 60 minutes later. The serum glucose level was measured and none of the patients had blood glucose levels exceeding 130 mg/dL before the injection of FDG. Two combined PET-CT in line system (BiographDUO, Biograph Truepoint; Siemens Medical Solutions, Knoxville, TN) was used to acquire the dataset.

PET-CT scans were interpreted by experienced nuclear medicine physicians. The maximum standard uptake value (SUVmax) of the suspected lesions on PET-CT was recorded. PET-CT findings after induction chemotherapy were compared with those of initial PET-CT. Mediastinal blood pool activity is recommended as the reference background activity to define PET positivity for a residual mass. Metabolic responses were assessed and classified as complete if lesions were hypermetabolic at baseline and residual tumoral uptake of SUVmax < 2.5 or incomplete if lesions were hypermetabolic at baseline and residual tumoral uptake of SUVmax > 2.5.^8^

### Chemotherapy and Surgery

The patients received 2 to 4 cycles of induction chemotherapy (cisplatin, 75 mg/m^2^ and docitaxel, 75 mg/m^2^ on day 1, every 21-day intervals). All patients referred to thoracic surgeon for curative resection that was schedule to take place 4 to 8 weeks after the completion of induction chemotherapy.

### Statistical Analysis

The sensitivity, specificity, accuracy, and predictive value of PET-CT were calculated using the standard definitions. The tumor was pathologically staged according to the American Joint Committee on Cancer (AJCC) criteria (7th edition). Overall survival (OS) was defined as the time from the start of chemotherapy to death from any cause. Relapse-free survival (RFS) was defined as the time from the start of chemotherapy to any type of recurrence and death. Survival distributions were calculated by the Kaplan-Meier method and compared using the log-rank test.

## RESULTS

Patient characteristics are shown in Table 1. The median age of the patients was 59 years (range, 46–75 years). Most of tumors were right-sided (66.7%). Pathology reports of the patients revealed adenocarcinoma in 20 patients, large cell carcinoma in 1, and squamous cell carcinoma in 21. Two patients had clinical T1, 20 patients had clinical T2, 15 patients had clinical T3, and 5 patients had clinical T4 tumor. In all patients, N2 disease was confirmed by mediastinoscopy.

**TABLE 1:**
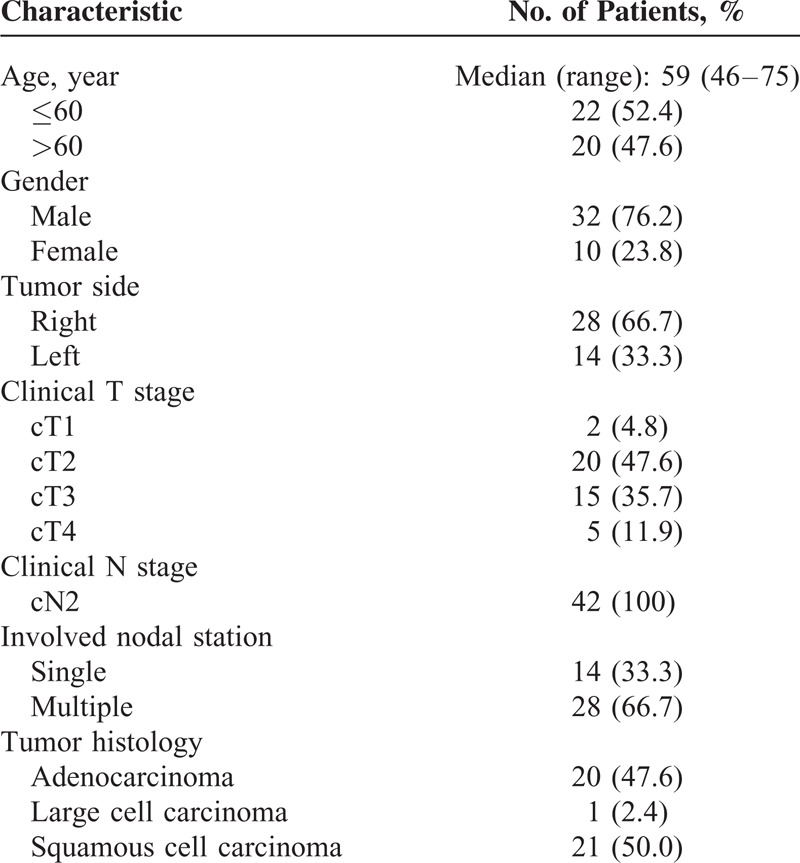
Patient Characteristics (n = 42)

### Comparison of cTN and ypTN Stage

Of the 42 patients, 12 patients received 2 cycles of induction chemotherapy with docitaxel and cisplatin, 23 patients received 3 cycles, and 7 patients received 4 cycles. Thirty nine (92.9%) patients underwent lobectomy and 3 (7.1%) patients underwent bilobectomy after induction chemotherapy. The clinical and pathologic stage after surgery are shown and compared in Table 2. Four (9.5%) of 42 patients had pathologically complete response (ypT0N0) of the primary tumor and nodal disease after preoperative chemotherapy. Downstaging of the primary tumor was observed in 22 (52.4%) of 42 patients and nodal downstaging was observed in 28 (66.7%) of 42 patients.

**TABLE 2:**
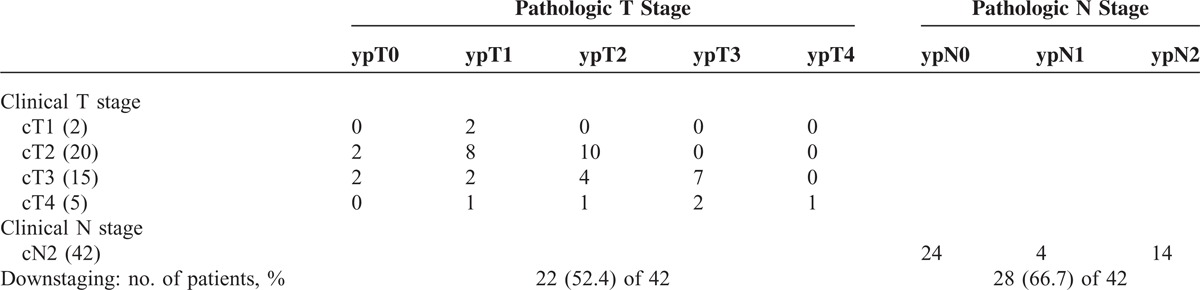
Comparison of Prechemotherapy and Postoperative T and N Stage

### Comparison of ycTN and ypTN Stage

The postchemotherapy and pathologic stage are shown and compared in Table 3. The accuracy of PET-CT in restaging of the primary tumor after induction chemotherapy was 50.0%. Eighteen (42.8%) of 42 patients were underestimated ycT stage, and 3 (7.1%) of 42 patients were overestimated ycT stage by PET-CT scan. The accuracy of PET-CT in restaging of the nodal disease was 71.4%. Six (14.3%) of 42 patients were underestimated ycN stage, and 6 (14.3) of 42 patients were overestimated ycN stage by PET-CT scan comparing the pathologic tumor and nodal staging.

**TABLE 3:**
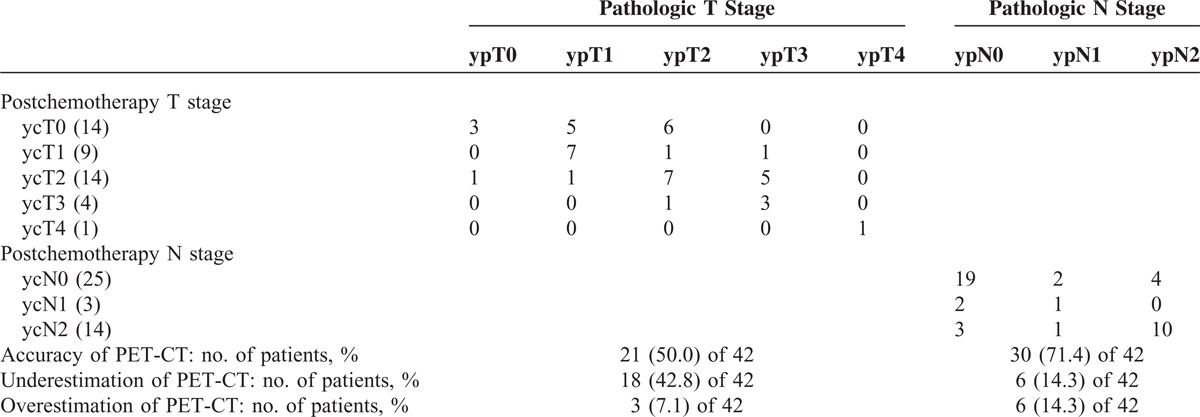
Comparison of Postchemotherapy and Postoperative T and N Stage

The sensitivity of PET-CT for the detection of the residual lymph node after induction chemotherapy was 70.6%, the specificity was 76.0%, and negative and positive predictive values were 79.2% and 66.7%, respectively.

### Recurrence and Survival

**FIGURE 1:**
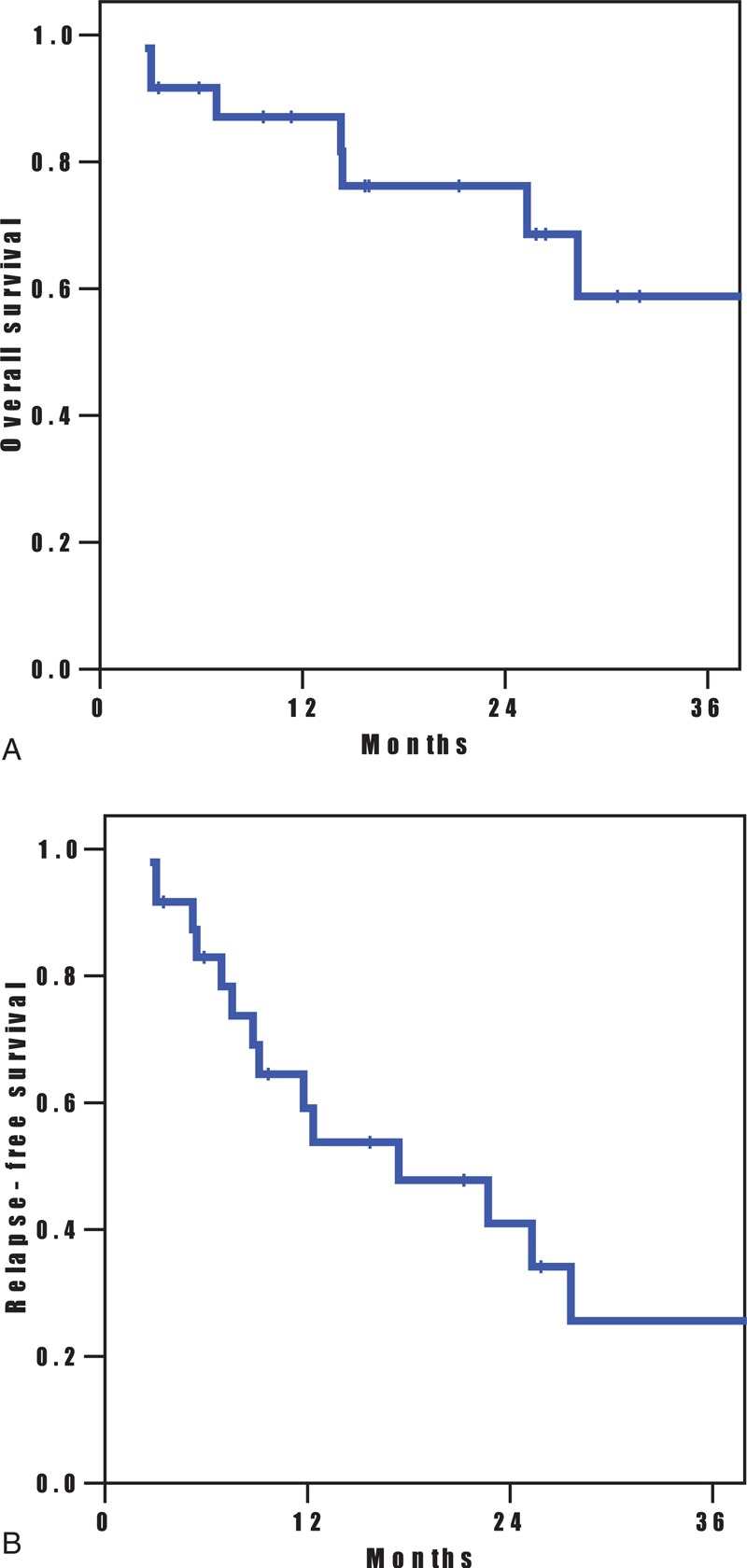
(A) Overall survival (OS) and (B) relapse-free survival (RFS) of the 42 patients who received induction chemotherapy with or without radiation and curative surgery were shown. The OS and RFS at 2 years were 68.5% and 40.9%, respectively.

**FIGURE 2:**
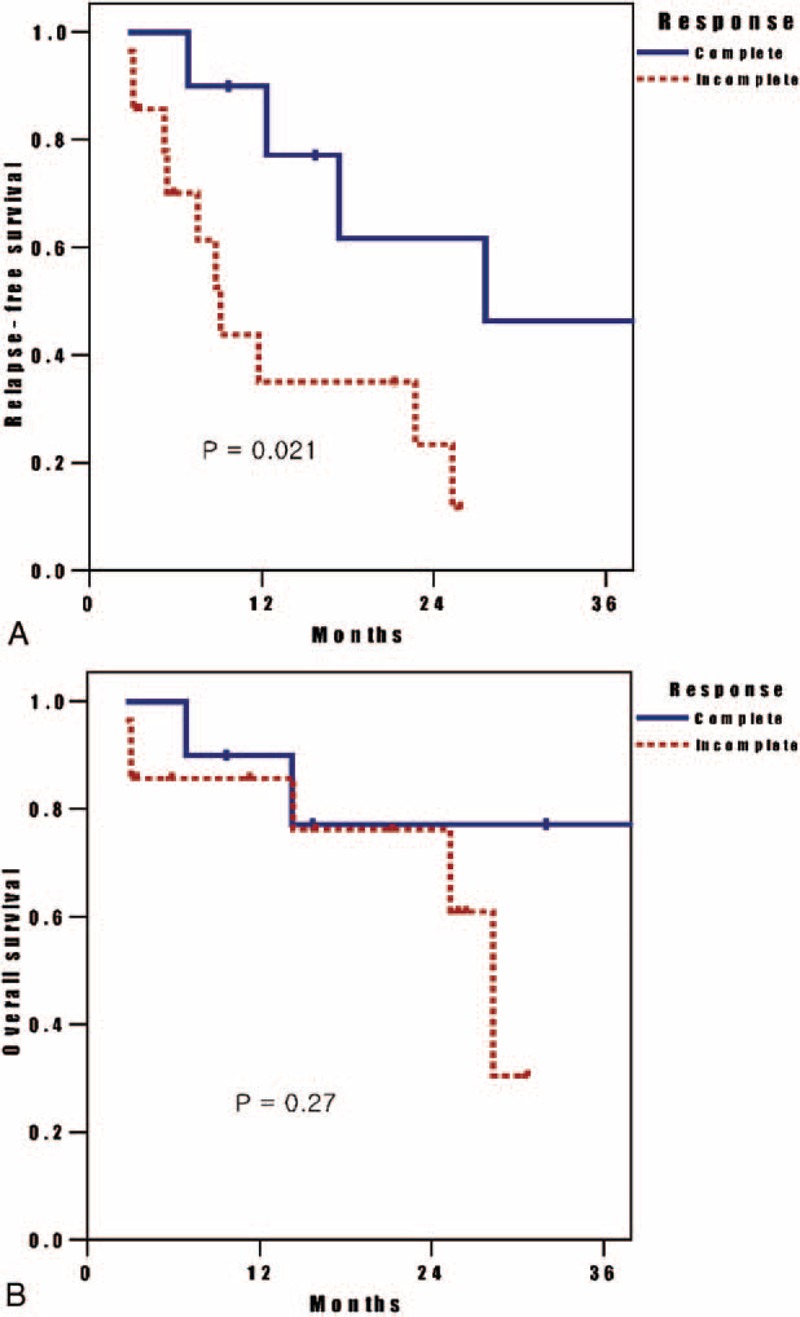
(A) Relapse-free survival (RFS) and (B) overall survival (OS) among the 42 patients according to the metabolic response on PET-CT (complete vs incomplete) were shown. The complete responders (ycT0N0M0) had a higher rate of RFS at 2 years than did the incomplete responders, and the difference was significant (61.7% vs 23.3%, *P* = 0.021). However, 2-year OS rates between complete and incomplete responders were not significantly different (77.1% vs 76.1%, *P* = 0.27).

During the median follow-up period of 28 months, 13 patients experienced locoregional relapse, and 7 patients occurred distant metastases. The remaining patients were alive and free of disease at the last follow-up time. The OS and RFS of the 42 patients who received induction chemotherapy and curative surgery were shown in Figure 1. The 2-year OS rate was 68.5%, and 2-year RFS rate was 40.9%. The OS and RFS of the 42 patients according to the response on PET-CT after induction chemotherapy were shown in Figure 2. Median time to recurrence was significantly longer in patients with complete response (ycT0N0M0) on PET-CT when compared to incomplete response (28.3 vs 9.1 months, respectively, *P* = 0.021). Two-year RFS rates between complete and incomplete responders were 61.7% and 23.3%, respectively. However, there is no significant difference in median survival time between complete and incomplete responders on PET-CT after induction chemotherapy (*P* = 0.27). Two-year OS rates between complete and incomplete responders were 77.1% and 76.1%, respectively.

## DISCUSSION

For patients with clinical stage IIIA-N2 NSCLC, multidisciplinary team approach is recommended before definitive treatment, and concurrent chemoradiotherapy is the treatment of choice according to the NCCN guideline.^3^ However, induction chemotherapy followed by surgical resection is another treatment option for operable advanced disease and is still under investigation.^4^

The exact response evaluation after induction chemotherapy in locally advanced NSCLC may be essential for determining surgery or chemoradiation after induction chemotherapy and predicting the prognosis of locally advanced NSCLC patients. However, restaging after induction chemotherapy assessed by chest CT scan alone has its limitations, and the use of PET-CT has shown promising results for assessing the response since it provided additional metabolic information.^9^

**FIGURE 3:**
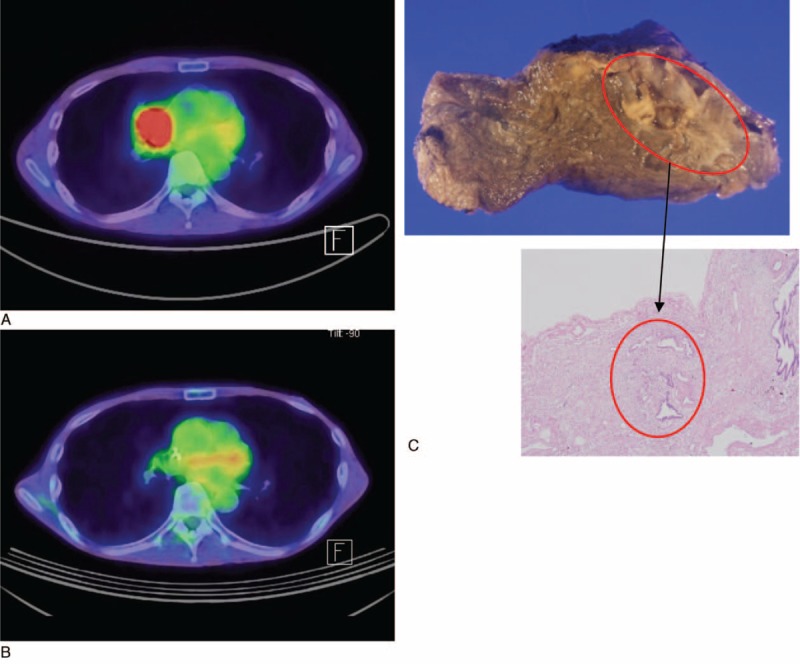
(A) A 63-years-old man had a hypermetabolic mass in right middle lobe. Adenocarcinoma was confirmed by a bronchoscopic examination. (B) After 3 cycles of induction chemotherapy, he had complete tumor response on PET-CT. One month later, he underwent a lobectomy of right middle lobe. **(**C**)** However, there was a remnant viable tumor on the pathologic examination (hematoxylin-eosin, original magnification ×40).

The response evaluation after induction chemotherapy on PET-CT has been the subject of recent trials in some types of tumors, including NSCLC. In lymphoma, PET-CT scan is a standard method to monitor response to chemotherapy and radiotherapy for its high accuracy and sensitivity, and complete response on PET-CT after chemotherapy or radiotherapy correlates with a better prognosis.^10^ In esophageal cancer, changes in tumor metabolic activity after preoperative chemoradiotherapy are significantly correlated with tumor response and patient survival.^11^ In our series, sensitivity and specificity of PET-CT in restaging of the nodal disease after induction chemotherapy were 70.6% and 76.0%, respectively. These were in accordance with other studies which reported 60% to 70% rate of sensitivity and 60% to 90% rate of specificity of PET-CT scan after induction therapy.^12^ This rather low sensitivity and specificity may result from small residual tumor nests that are surrounded by fibrosis which are difficult to detect (Figure 3). Restaging of nodal disease on PET-CT after induction chemotherapy has been associated with 20.8% of false positivity in our analysis. Macrophage could infiltrate the marginal areas surrounding necrotic area of the tumor, and macrophage infiltration is 1 factor which induced high FDG uptake after induction chemotherapy.^13^ In vitro model, the FDG signal could be increased up to 30% by the macrophage/monocyte infiltration.^14^ FDG accumulation in inflammatory lesions as well as in tumors reduces the diagnostic sensitivity of PET-CT in oncology.

In our study, 28 (66.7%) of 42 patients experienced nodal downstaging after induction chemotherapy, and at median follow-up period of 28 months, RFS time was significantly longer in complete responders on PET-CT when compared with incomplete responders (28.3 vs 9.1 months, respectively, *P* = 0.021). Our results agree with those of previous studies which demonstrated a positive and significant correlation between PET-CT findings and survival in postchemotherapy staging of NSCLC.^9,15–17^ In Decoster et al,^9^ PET-CT response is highly predictive for progression-free and OS in patients with inoperable NSCLC treated with induction chemotherapy. However, we failed to demonstrate the significant difference of the overall survival time between complete and incomplete responders on PET-CT after induction chemotherapy (*P* = 0.27).

Despite the very significant association between PET-CT response and RFS in our study, we acknowledge that our series had a number of limitations. First, our study should be understood in view of the inherent biases of a retrospective study design. We did not evaluate 3 NSCLC patients who did not evaluate the postchemotherapy PET-CT scan and patients who had distant metastases; thus, we deliberately did not evaluate several cases with advanced NSCLC who had received induction chemotherapy and surgery. Second, the enrolled number of patients in our trial is just 42 cases, thus the analytic power is limited.^18^

On the basis of these results, complete response on PET-CT after induction chemotherapy with or without radiation was a prognostic factor for RFS in stage IIIA-N2 NSCLC patients who underwent curative resections. However, postchemotherapy value of PET-CT should be interpreted with caution due to its unacceptable sensitivity and accuracy.
